# Reuse of Boron Waste as an Additive in Road Base Material

**DOI:** 10.3390/ma9060416

**Published:** 2016-05-26

**Authors:** Yutong Zhang, Qinglin Guo, Lili Li, Ping Jiang, Yubo Jiao, Yongchun Cheng

**Affiliations:** 1 Jilin Provincical High Class Highway Construction Burean, Changchun 130033, China; zhangyutong0304@gmail.com; 2 College of Transportation, Jilin University, Changchun 130025, China; jiaoyb@jlu.edu.cn (Y.J.); chengyc@jlu.edu.cn (Y.C.); 3 School of Civil Engineering, Hebei University of Engineering, Handan 056038, China; 4 Department of Student’s Affairs, Hebei University of Engineering, Handan 056038, China; Lill@hebeu.edu.cn; 5 School of Civil Engineering, Shaoxing University, Shaoxing 312000, China; jiangping@usx.edu.cn

**Keywords:** boron waste, stabilized material, road base, mechanical properties, drying shrinkage

## Abstract

The amount of boron waste increases year by year. There is an urgent demand to manage it in order to reduce the environmental impact. In this paper, boron waste was reused as an additive in road base material. Lime and cement were employed to stabilize the waste mixture. Mechanical performances of stabilized mixture were evaluated by experimental methods. A compaction test, an unconfined compressive test, an indirect tensile test, a modulus test, a drying shrinkage test, and a frost resistance test were carried out. Results indicated that mechanical strengths of lime-stabilized boron waste mixture (LSB) satisfy the requirements of road base when lime content is greater than 8%. LSB can only be applied in non-frozen regions as a result of its poor frost resistance. The lime–cement-stabilized mixture can be used in frozen regions when lime and cement contents are 8% and 5%, respectively. Aggregate reduces the drying shrinkage coefficient effectively. Thus, aggregate is suggested for mixture stabilization properly. This work provides a proposal for the management of boron waste.

## 1. Introduction

**Figure 1:**
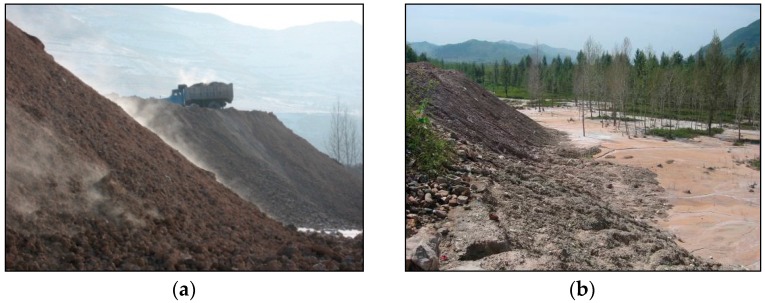
Effects of boron waste on the environment. (**a**) Air storage; (**b**) Waste diffusion.

Boron waste is discharged in the production of borax pentahydrate, borax decahydrate, anhydrous borax, *etc.*, and its amount increases year by year. In China, its reserve has reached 35 million tons, and emission still grows at a rate of 1.6 million tons per year [1]. Generally, it is open-air stacked near the ore. The waste containing boron oxide will pollute the earth and water because the hazardous materials will be transmitted to groundwater resource from where it is disposed [2]. Its environmental impacts are shown in Figure 1.

It can be seen from Figure 1 that the disposal and storage will require additional costs, which cannot be negligible in quantity [2]. Large areas have to be allocated for disposal. Thus, waste storage causes environmental pollution and economical loss [3]. The utilization of waste is helpful for environment protection and saving costs. To this end, it has been reused as building material in several countries. Reused technology has also been studied by many scholars.

Ozdemir *et al.* [3] added boron waste into the clinker and gypsum of cement. Their results showed that the mechanical properties of cement mortar decreased with the increase in waste. Optimum contents for two kinds of waste were 5% and 10%, respectively. Palomo *et al.* [4] studied the effect of boron waste on the hardening process of a new alkali-activated fly ash matrix. Results implied that the waste hardly modified the mechanical strength of the matrix. The boron diffusion rate of activated fly ash-lime matrix was 100 times less than that of ordinary cement-lime matrix. Boncukcuoglu *et al.* [5] investigated the influence of thromel sieve waste on mechanical performances of Portland cement and concrete. It was demonstrated that the setting time of concrete decreased when thromel sieve waste (TSW) was added. Compressive and tensile strengths decreased with the increase in TSW. The suggested content of TSW was 25% of the weight of the cement. Boron waste was reused as a cement additive in these studies. Boron waste improved the mechanical and physical properties of the cement and the fly ash-lime matrix. The maximum dosage of this waste for the cement is 25%. However, reused amounts of it is small because aggregate is the main phase of concrete.

For the purposes of sustainable development and industrial ecology, boron waste has been gradually used in the concrete and brick. Topçu *et al.* [6] investigated impact of boron waste on the workability and strength of concrete. They indicated that it slightly influenced the workability. However, mechanical strength was reduced. The strength of concrete saw no obvious change when its content was less than 10%. Moreover, this waste improved the durability of the concrete. Abalı *et al.* [2] prepared lightweight brick with boron waste. The effects of waste, the burning rate, and the temperature on the performance of the brick were investigated. They suggested that it could not be used in structural brick since the waste brought about crashing during the firing process. Uslu *et al.* [7] also pointed out that brick quality improved when the waste percentage was less than 30%. Results of a study by Özdemir *et al.* [8] demonstrated that the compressive and flexural strengths of modified mortar were higher than the control sample, while waste content was less than 1.5%. The strength of modified mortar was nearly equal to the strength of that of the control when its content was 2.5%–5.0%. Waste has a small effect on the durability of mortar. In addition, Kavas *et al.* [9] utilized this waste as a fluxing agent in the production of red mud brick. Their results implied that the firing temperature decreased. The compressive property of brick increased when waste content was over 15 wt %. Recently, they also utilized the waste to produce artificial lightweight aggregate (LWA). It was suggested that the sieve and dewatering boron waste (SBW and DBW) manufactured lightweight aggregate [10].

It can be seen that reuse of boron waste in brick and concrete increases the managing efficiency. For concrete, its content should be less than 15%. For brick and lightweight aggregate, its amount should be less than 30%. Remarkably, the strength of concrete and brick obviously decreases when the content exceeds the limited value. This means that boron waste cannot be massively applied in high strength material. Therefore, further research is needed to explore other reuse methods. Fortunately, Modarres *et al.* [11] proposed that pavement engineering could result in a change that is friendly to the environment. Reuse of waste material in pavement could solve the environmental pollution. As we all know, the lime–cement-stabilized granular material is always used for road base or sub-base. Moreover, the demanded quantity of stabilized material is great due to the enormous quantity of the road. Herein, impacts of boron waste on the environment and the economy will be solved if it is successfully used in road base.

In order to find a good stabilized method, many studies have been performed in recent years. Qian *et al.* [12] investigated the performances of cement-stabilized granite mill tailings for road sub-base. Results showed that the strength and stiffness of the cement-stabilized mixture met the structural requirements of pavement. A better curing was required in order to avoid shrinkage cracking. The suggested cement content was 4%–6%. Joel *et al.* [13] used cement to stabilize the reddish-brown lateritic soil in Nigeria. They found that the plasticity index of laterite decreased with the increase in sand and cement. Optimum moisture content (OMC) increased with cement but decreased with the sand. Jauberthie *et al.* [14] studied the geotechnical characteristics of lime and cement-stabilized estuarine silt. They suggested that the unconfined compressive strength (UCS) and the California bearing ratio (CBR) increased with the increase in lime and cement. The lime–cement-treated mixture was superior to that treated by lime only. This means that lime and cement are good binding materials for stabilized soils, crush stones, and crushed masonry. In other words, these stabilized methods may be applicable to boron waste. The feasibility of these methods should also be verified systemically.

Based on the above analysis, we can find that boron waste has a significant impact on the environment. Its reuse is helpful for protecting the environment. In order to increase the reused amount as much as possible, it was reused in the road base material in this study. Lime and cement were used to stabilize this waste. The stabilized waste mixture was prepared in a laboratory. Specimens were then made according to the standards JTG E51-2009 [15], and the physical, chemical, and mechanical performances of the stabilized waste mixture were tested and evaluated.

## 2. Materials

**Figure 2:**
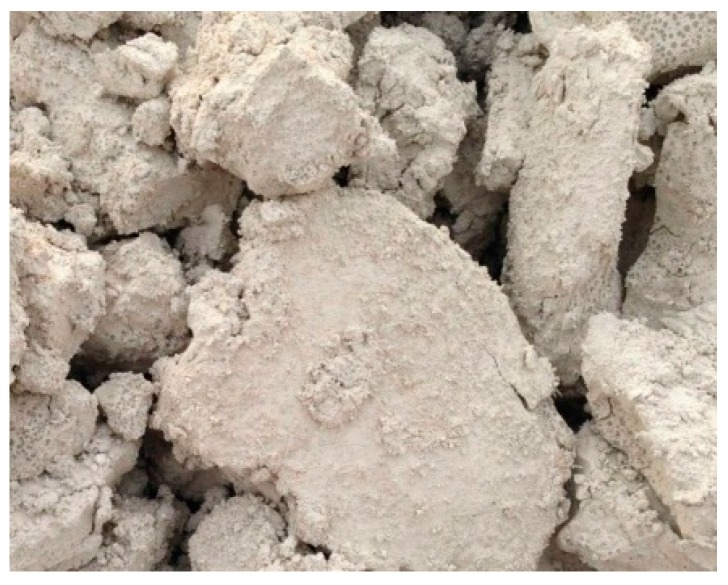
Boron waste.

Boron waste, which was discharged in the production of borax, was used in this study. Its color is light brown, as shown in Figure 2.

Basic properties of boron waste are given in Table 1. A laser diffraction particle size analyzer was employed to determine its size distribution. Results are shown in Table 2. The non-uniformity coefficient (*C_u_*) and coefficient of curvature (*C_c_*) are also given in Table 2.

The content of effective calcium oxide and magnesium oxide in hydrated lime is 60.4%. Ordinary Portland cement with a level of 42.5 MPa was used in this study. The properties of the cement are listed in Table 3.

**Figure 3:**
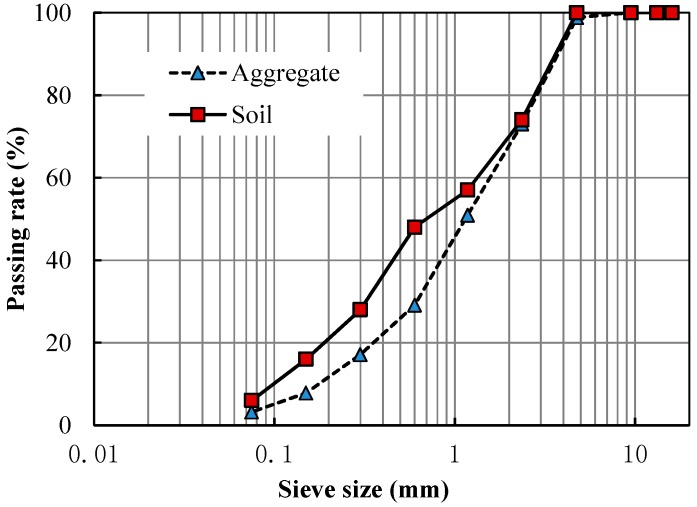
Gradations of the aggregate and soil.

Limestone aggregate and soil were selected for experiments. The apparent density of aggregate was 2.677 g/cm^3^. The properties of the soil were tested according to the Test Methods of Materials Stabilized with Inorganic Binders for Highway Engineering of China (JTG E51-2009) [15], as given in Table 4. Gradations of the aggregate and soil are shown in Figure 3.

## 3. Experimental Methods

### 3.1. Compaction Test

Hydrated lime and cement have been widely used to stabilize soil and granular material [16,17,18]. Therefore, they were selected to stabilize boron waste in this study. In order to utilize the boron waste as much as possible and reduce the project cost, properties of hydrated lime-stabilized boron waste were investigated firstly. In China, the suggested content of lime was less than 12% of the weight in accordance with the Specifications for Design of Highway Asphalt Pavement (JTG D50-2006) [19]. Herein, five lime contents (3%, 5%, 8%, 10%, 12%) were selected for experiment. The stabilized mixture was prepared in laboratory, and it was then well-sealed and placed for 12 h in a container. A compaction test is often used to determine the optimum moisture content (OMC) in the road base design. Thus, hydrated lime-stabilized boron waste was compacted in the laboratory according to ASTM D 698 [20]. Finally, the relationship between moisture content and dry density was determined.

### 3.2. Unconfined Compressive Test

Unconfined compressive strength (UCS) is an important index of geo-materials [17,21,22]. An unconfined compressive test is often used to determine the compressive strength under the unconfined state. A higher UCS value means a better engineering performance. Thus, it was used to determine the optimum proportion of the road base mixture. In the laboratory, 18 cylinder specimens were prepared using the static compaction method. As specified in JTG E51-2009 of China [15], the diameter and height of the specimens were both 50 mm. The specimens were grouped and cured for 7 and 28 days, respectively. The curing temperature and relative humidity were 20 ± 2 °C and 95%, respectively. It should be stated that specimens were saturated for 24 h on the final day of curing period.

### 3.3. Indirect Tensile Test

An indirect tensile strength (ITS) can be used to evaluate the anti-cracking performance of geo-materials [23]. Therefore, an indirect tensile test was conducted on the stabilized mixture in this study. The size of the cylinder specimen was Ф50 mm × 50 mm. Specimens were cured for 90 days before the test. The width of the load strip was 6.35 mm. The inside radius of the strip was 25 mm. The selected loading rate was 1 mm/min. On the basis of the test result, the indirect tensile strength (ITS) and the ultimate tensile strain (UTS) can be calculated by the following equations:

(1) $$ITS=\frac{2P}{\pi dH}\left(\sin2\alpha -\frac{a}{d}\right),\;\text{and}$$

(2) $$UTS=\frac{ITS}{E}$$

where *P* is the ultimate load, N; *H* is the height of specimen, mm; and *d* is the diameter of specimen, mm. *a* is the width of loading strip, mm. α is the center angle corresponding to half width of the strip, rad. *E* is resilient modulus, MPa.

### 3.4. Modulus Test

Baghini *et al.* [21] indicated that a resilient modulus was an important parameter for the road design and analysis. It is a basic parameter for flexural stress analysis in the road design. Therefore, the resilient modulus of stabilized boron waste mixture was tested. Cylinder specimens with a size of Ф100 mm × 100 mm were prepared in the laboratory firstly. Then, these specimens were cured for 90 days. The curing temperature and relative humidity were 20 ± 2 °C and 95%, respectively. Specimens were saturated for 24 h on the final day of the curing period before test. According to the Test Methods of Materials Stabilized with Inorganic Binders for Highway Engineering of China (JTG E51-2009) [15], a maximum pressure of 0.72 MPa was employed in this test. It was divided for six stages, the applied pressure were 0.12, 0.24, 0.36, 0.48, 0.60, and 0.72 MPa, respectively. For every stage, the deformed value *l*_1_ was recorded when the specimen was loaded for 1 min. Then, the pressure was unloaded. The value *l*_2_ of the gauge was recorded when the specimen was unloaded for 0.5 min. Then, the next pressure was subsequently applied. This process was repeated step by step until all stages were finished. The resilient modulus (*E*) can be computed by the following equations.

(3) $$l=\begin{array}{ccc} \bar{l_{1i}} & - & \bar{l_{2i}} \end{array},\;\text{and}$$

(4) $$E=\frac{pH}{l}$$

where *P* is the maximum pressure, MPa; *H* is the height of specimen, mm; and *l*, $\bar{l_{1i}}$, $\bar{l_{2i}}$ are resilient deformations of specimen, mm. i=1,2,⋯,6.

### 3.5. Drying Shrinkage Test

**Figure 4:**
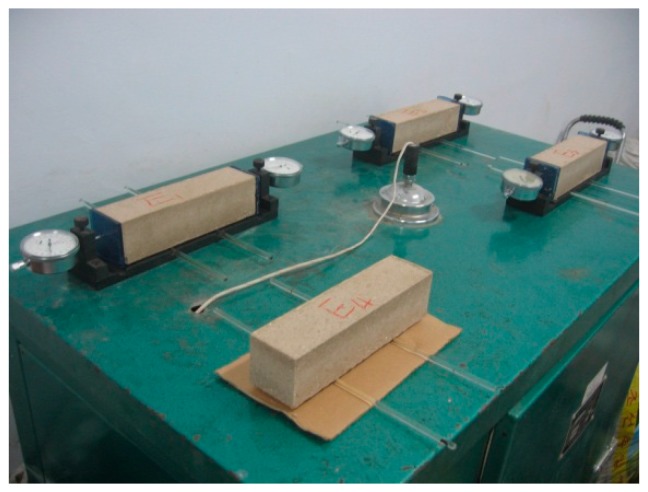
Drying shrinkage test.

Pozo-Antonio [17] and Idiart *et al.* [24] indicated that consequently drying shrinkage caused micro-cracks around aggregate. This will result in the decrease in mechanical properties and road base cracking. In this study, the drying shrinkage properties of the stabilized boron waste mixture were investigated in this study. Beam specimens with a size of 50 mm × 50 mm × 200 mm were prepared in room according to Standard JTG E51-2009 [15]. Two glass sheets were attached to both ends of the specimen in order to obtain precise shrinkage deformation. Then, the beam was placed on the glass support to ensure free shrinkage. The shrinkage deformation and the mass of the specimen were recorded every day because the shrinkage deformation was very small within a short period. The test was terminated until the mass did not change with time. Then, the specimens were dried to constant masses in an oven. The process of the drying shrinkage test was shown in Figure 4.

In this study, the drying shrinkage coefficient was employed to evaluate the drying shrinkage performance. It can be calculated by the following equations:

(5) $$\omega_{i}=(m_{i}-m_{i-1})/m_{p}$$

(6) $$\delta_{i}=(\sum_{j=1}^{4}X_{i,j}-\sum_{j=1}^{4}X_{i-1,j})/2$$

(7) $$\varepsilon_{i}=\delta_{i}/l,\;\text{and}$$

(8) $$\alpha_{di}=\frac{\varepsilon_{i}}{\omega_{i}}$$

where $\omega_{i}$ is the rate of moisture loss, %; *m_i_*, *m_i_*_−1_ are the masses of specimen on the *i*th day and the *(i* − 1*)*th day, respectively, g; *m_p_* is the dry mass of specimen, g; δ*_i_* is the drying shrinkage deformation on the *i*th day, mm; *X_i_,_j_* and *X_i_*_−1_*,_j_* are the values of the *j*th gauge on the *i*th day and *(i* − 1*)*th day, mm; ε*_i_* is the drying shrinkage strain on the *i*th day, %; *l* is the initial length of specimen before test, mm; α*_di_* is the shrinkage coefficient on the *i*th day, %.

### 3.6. Frost Resistance Test

Kelestemur *et al.* [25] and Jafari *et al.* [26] have indicated that the strength of stabilized materials decreased under the action of freeze–thaw cycles. This means that the action of freeze–thaw has an adverse impact on the mechanical properties of stabilized material. The frost resistance of base materials is necessary to ensure the bearing stability of the road. For this purpose, the frost resistance of lime and lime–cement-stabilized boron waste mixture was investigated in this study. Cylinder specimens with a size of Ф50 mm × 50 mm were prepared for a frost resistance test in accordance with JTG E51-2009 [15]. These specimens were cured for 28 days under the same conditions as in the previous description. After the curing, specimens were saturated and frozen for 16 h at −18 °C. Then, specimens were thawed in the water at 20 °C for 8 h. The total number of the freeze–thaw cycle was 5. UCS’s of specimen were tested before and after freeze–thaw cycles. The frost resistance index (FRI) can be calculated with the following equation:

(9) $$FRI=\frac{R_{FT}}{R_{C}}\times 100,$$

where *FRI* is the indicator that represents the frost resistance—a high value means a good frost resistance. *R_FT_* is the unconfined compressive strength of specimen after five freeze–thaw cycles, MPa; and *R_c_* is the unconfined compressive strength of control specimen, MPa.

### 3.7. FIIR Test

Palomo *et al.* [4] indicated that XRD was suitable for the study of Portland cement matrices; however, for matrices containing boron waste, a FTIR test is better since it gives a major vitreous phase together with some crystalline quartz, mullite, and hematite. Therefore, cylinder specimens with a size of Ф50 mm × 50 mm were prepared and cured for 90 days. Then FTIR test was conducted on the surface of specimens. Curing temperature and relative humidity were 20 ± 2 °C and 95%, respectively.

## 4. Results and Discussion

### 4.1. Properties of Lime-Stabilized Boron Waste

In order to save the construction cost, lime was firstly used to stabilize the boron waste alone. The feasibility of the lime-stabilized boron waste (LSB) for the road base was investigated. The optimum moisture content (OMC) and maximum dry density (MDD) for every proportion were determined. Kweon *et al.* [27] indicated that frost action had a significant effect on the performance of road base in seasonal freezing regions. Therefore, frost resistance of stabilized material cannot be ignored in these areas. A freeze–thaw test was conducted in the laboratory according to JTG E51-2009. The unconfined compressive strength (UCS) and frost resistance index (FRI) for lime-stabilized boron waste were obtained. In order to evaluate the effects of lime, a statistical analysis of variance (ANOVA) method was applied to investigate the significance. The significance level (α) was 0.05. F-tests were performed based on a confidence level 95%. Results are listed in Table 5.

**Figure 5:**
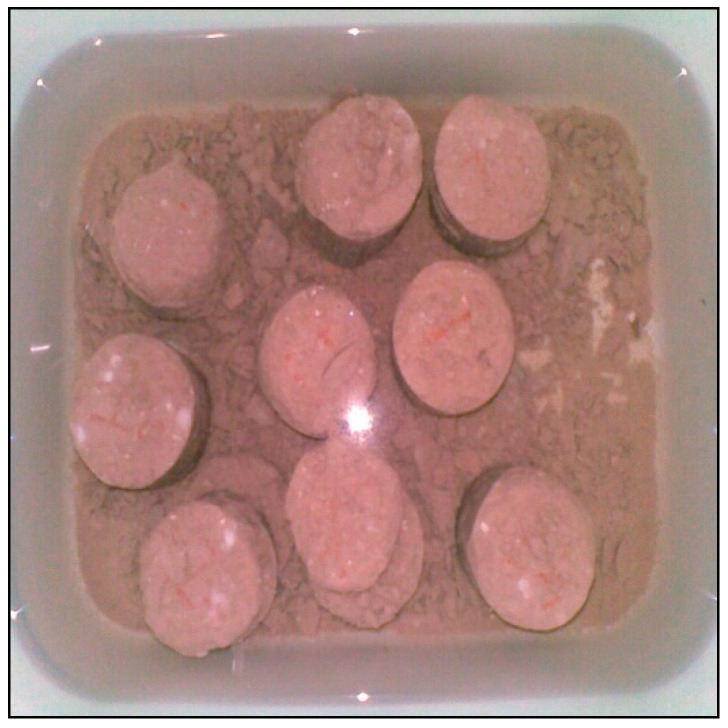
Specimens after the freeze–thaw test.

It can be seen from Table 5 that the optimum moisture content (OMC) of the stabilized mixture increases with lime, but the maximum dry density (MDD) decreases. This agrees with the results of Edeh *et al.* [28]. The unconfined compressive strength (UCS) increases with the lime content and curing time. However, UCS’s are both not available for curing 7 and 28 days when lime content is 3%. This means that the cementitious phases such as hydrated silicate, calcium hydroxide crystals, and calcium carbonate are insufficient to form the bearing structure in the stabilized mixture when lime content is low. This causes damage to the LSB. Furthermore, the suggested UCS (7 days) of lime-stabilized material is greater than 0.8 MPa according to the Specifications for Design of Highway Asphalt Pavement of China (JTG D50-2006) [19]. For the sub-base, it should be greater than 0.6 MPa. Therefore, LSB can be used to build the sub-base when the lime content is greater than 5%. Lime content should be greater than 8% for the road base. Results of the statistical ANOVA demonstrate that the lime content has a significant influence on OMC and UCS. The effect of lime content on MDD is not significant. This may be induced from the experimental accuracy. Specimens after the freeze–thaw test are shown in Figure 5.

As shown in Table 5 and Figure 5, specimens were destroyed after four freeze–thaw cycles. Frost resistance indexes (FRI) are not available for all kinds of LSB. This means that the lime-stabilized boron waste does not satisfy the anti-freezing requirement of the road base or sub-base. LSB is only suitable for non-frozen regions. The other stabilized method should be sought to improve the frost resistance of LSB if it is used in frozen regions.

### 4.2. Properties of Lime–Cement-Stabilized Boron Waste Mixture

Balen *et al.* [29] proposed that lime mortar had a better durability than the cement-based mortar, although the cement mortar had a better compressive strength. Arandigoyen *et al.* [30] pointed out that the mechanical strength of cement mortar decreased when little lime was added. The mechanical strength of lime mortar increased when cement content was less than 40%. In addition, Papayianni *et al.* [31] proposed that aggregate could improve the moisture and frost resistance. Therefore, lime and cement were both used to stabilize boron waste in this study. Lime–cement-stabilized boron waste and soil (LCBS) and lime–cement-stabilized boron waste and aggregate (LCBA) were prepared in the laboratory. Six proportions were selected for the experiment in accordance with JTG D50-2006 [19]. The compaction test was conducted to determine the OMC and MDD of the different mixtures. Average value (AV) and standard deviation (SD) of the compaction test are given in Table 6.

As listed in Table 6, optimum moisture contents (OMC) decrease with the increase in boron waste for LCBS. On the contrary, optimum moisture contents (OMC) of LCBA rise with boron waste. For LCBS and LCBA, the changing trends of OMC are different. It agrees with the results of Edeh *et al.* [28] and Modarres *et al.* [18]. Edeh *et al.* [28] proposed that more water was required to lubricate the entire matrix of the mixture when the surface area of particles increased. Modarres *et al.* [18] also thought that the finer the gradation was, the more moisture content there was. Therefore, the variation in OMC can be attributed to the change in the particle surface area. For LCBS, the reduction of fine soil and increase in boron waste cause a decline in surface area as a whole. This leads to a low OMC. For LCBA, variations in boron waste and aggregate result in a rise in surface area. A higher OMC results. Additionally, their maximum dry densities (MDDs) also decrease with the increase in boron waste. Baghini *et al.* [21] thought that the change in MDD could be explained by the void formulation theory. Lade *et al.* [32] also indicated that the overall void ratio decreased until all voids were filled with small particles. Therefore, the change in MDD is induced by a mixed proportion. The void ratio is different at different gradations [33]. In other words, the gradation of the stabilized mixture has a significant effect on MDD.

**Figure 6:**
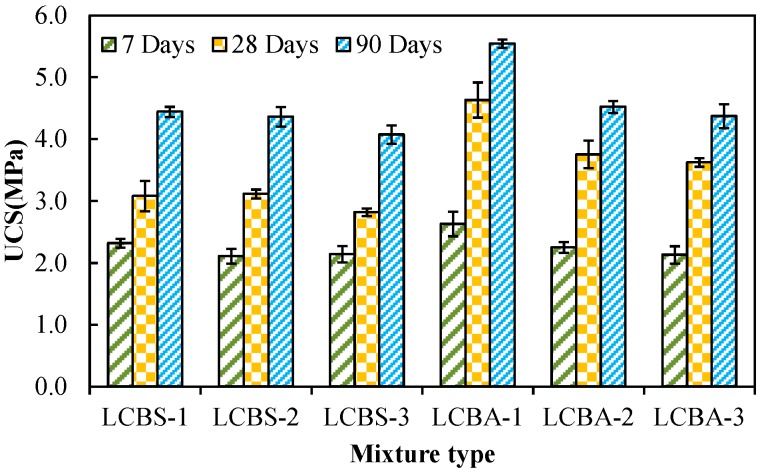
UCS of stabilized mixtures with different curing periods.

Unconfined compressive strength (UCS) is a common mechanical property, which has been used to determine the proportion of stabilized mixture. Therefore, the UCS’s of the stabilized mixtures were investigated. Three curing periods at 7, 28, and 90 days were selected for the experiment. Results are shown in Figure 6.

**Figure 7:**
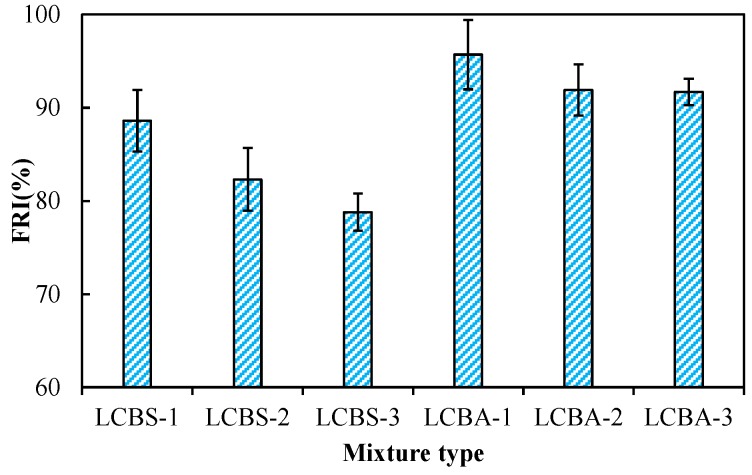
FRI of the stabilized mixture after curing for 28 days.

It can be seen from Figure 6 that UCS’s of stabilized mixtures are greater than 0.8 MPa when they were cured for 7 days. This means that the strengths satisfy the structrual requirement of the road base. UCS’s of all stabilized mixtures increase with curing time. This trend agrees with the results of Modarres *et al.* [18] and Taha *et al.* [34] and is attributed to the hydration reaction of cement and lime. In addition, UCS’s of LCBA are greater than that of LCBS. It can be inferred that aggregate has better reinforcing effect. However, the road base may also be damaged in the seasonal freezing region, although UCS satisfies the strength requirement [25]. Therefore, frost resistance of stabilized boron waste mixture needed to be evaluated. Results of the freeze–thaw test are shown in Figure 7.

As shown in Figure 7, FRIs are all greater than 70%. According to JTG D50-2006 [19], the frost resistance of the stabilized mixture meets the anti-freezing requirement. The FRIs of LCBA are all greater than that of LCBS. This implies that both LCBS and LCBA can be applied in seasonal freezing regions such as Jilin province in China. Jafari *et al.* [26] pointed out that fine particles that have poor cohesion were sensitive to freeze–thaw cycles. Freezing saturated big pores caused high internal stress. It was intolerable for weak-bounded particles. For stabilized material, disconnected pores are filled by a cementitious phase. This prevents the water migration from freezing, and ice lenses will not grow as much as those of untreated material. There are three reasons for good frost resistance. Firstly, the gradation of aggregate is coarser than that of the soil. The gradation of soil is coarser than boron waste. Aggregate and soil reduce the freezing susceptibility of the stabilized mixture. Secondly, lime and cement fill the pores in the stabilized mixture, and the water migration is prevented. Finally, lime and cement improve the cohesive properties of the stabilized mixture. Therefore, lime and cement are both suggested for a stabilized boron waste mixture in order to improve its frost resistance.

**Figure 8:**
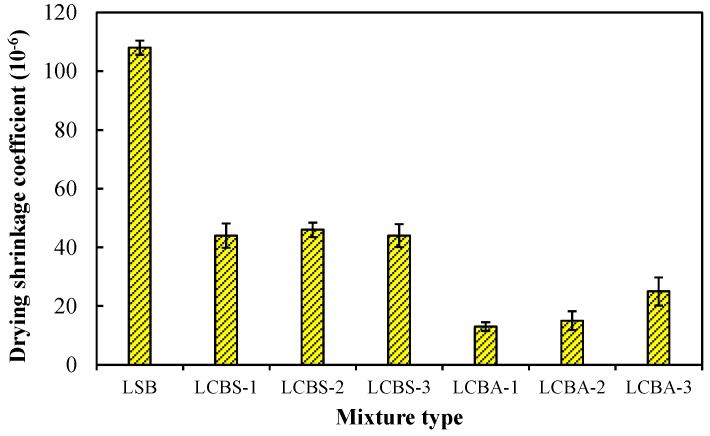
Drying shrinkage coefficient of stabilized boron waste mixture.

For a cementitious composite, Idiart *et al.* [24] indicated that the drying shrinkage of a matrix was restricted by aggregate and that it will lead to internal micro-cracking under certain conditions. Drying shrinkage cracks are detrimental to the road base. Consequently, the drying shrinkage performance of the stabilized mixture was investigated here. Chindaprasirt *et al.* [35] proposed that the shrinkage rate was very high at an early age. The shrinkage coefficient of this period was maximal. Therefore, the maximum shrinkage coefficient was used to evaluate drying shrinkage. Specimens that had been cured for 28 days were used for the drying shrinkage test. The maximum shrinkage coefficient was calculated using Equation (8). Results are shown in Figure 8.

As shown in Figure 8, the drying shrinkage coefficient of LSB is the highest. The coefficients of LCBS are greater than that of LCBA. The coefficient of LCBA-1 is the lowest among all mixtures. This may be attributed to the phase of soil and aggregate. It can be inferred that aggregate improves the drying shrinkage performance significantly. Thus, it is suggested that aggregate is used in stabilized boron waste material. In addition, Ceylan *et al.* [36] indicated that reflective cracks through HMA overlays had been an international problem for decades. Although reflective cracks do not reduce the structural capacity of a pavement generally, the subsequent ingress of moisture, the natural environment, and traffic can cause premature distress and even pavement failure [37]. Therefore, LCBA was more suitable for the road base in order to reduce the reflective cracks. Meanwhile, shrinkage deformation will result in tensile stress. The material will not crack if stress does not exceed the ultimate value. Thus, the tensile properties of stabilized material is important. In this study, the tensile properties of the stabilized boron waste mixture was investigated using an indirect tensile method. A resilient modulus (*E*) was obtained by a uniaxial compression test. The indirect tensile strength (ITS) and ultimate tensile strain (UTS) were calculated by Equations (1) and (2), respectively. Results are listed in Table 7.

It can be found from Table 7 that the resilient modulus (*E*) of LCBS increased with the increase in boron waste. For LCBA, the resilient modulus decreased with the increase in boron waste. Their trends are different. This is caused by the difference in the elastic properties of aggregate, boron waste, and soil. ITS and UTS decrease with the increase in boron waste. This means that boron waste has an adverse effect on the tensile properties. Besides, ITS of LCBA-1 is the highest among all of the mixtures. The ultimate tensile strain (UTS) of LCBS-1 is the highest. UTS of LCBA-1 decreases by 17.0% compared with that of LCBS-1. It seems that the decline in UTS is adverse to drying shrinkage, but the shrinkage coefficient of LCBA-1 is less than that of LCBS-1. Therefore, LCBA-1 may be a good proportion on the whole.

### 4.3. Characterization of Chemical Reaction

**Figure 9:**
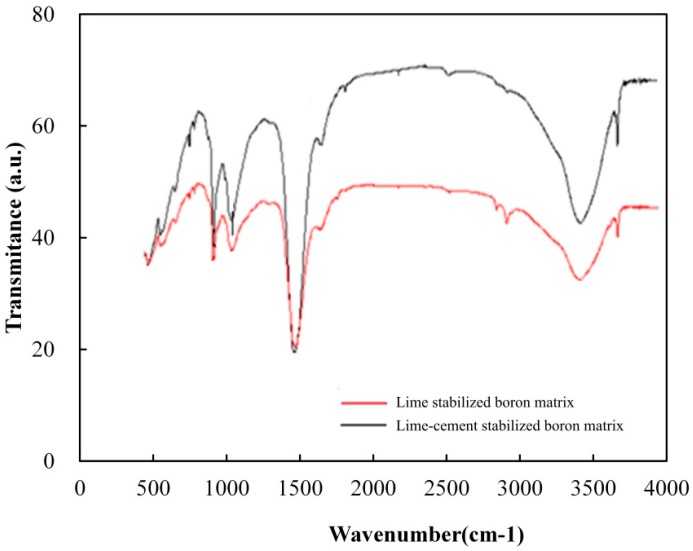
FTIR spectra of the lime-stabilized matrix and lime–cement-stabilized matrix.

FTIR spectra of the lime-stabilized matrix and the lime–cement-stabilized matrix were recorded with a Nexus 6700 spectrometer (Thermo Nicolet Corporation, Madison, WI, USA) in order to reveal their chemical reactions. Results of the FTIR test are shown in Figure 9.

As shown in Figure 9, no significant differences are observed between the lime-stabilized matrix and the lime–cement-stabilized matrix. The lime–cement-stabilized matrix has more hydrated silicate than the lime-stabilized one due to the addition of cement. According to the interpretation of bonds from the FTIR [4], the presence of the bond around 1450 cm^−1^ indicates the formation of MgCO_3_. The bonds around 1000 cm^−1^ correspond to Si–O and Al–O tension bonds. They are the characteristic bonds of the alkaline polymer. Bonds around 780 correspond to Si–O–Si bonds. Bonds around 3600 cm^−1^ indicate the presence of CaO or Ca(OH)_2_. SiO_2_ and Al_2_O_3_ in boron waste will be activated by CaO (or Ca(OH)_2_), and C–S–H and CaO (or Ca(OH)_2_) will form the C–S–H gel structure. The reaction equations can be written as follows:

$$2(3\text{CaO}\cdot {\text{SiO}}_{2})+6H_{2}O\to 3\text{CaO}\cdot 2{\text{SiO}}_{2}\cdot 3H_{2}O+3{\text{Ca(OH)}}_{2};$$

$$2(2\text{CaO}\cdot {\text{SiO}}_{2})+4H_{2}O\to 3\text{CaO}\cdot 2{\text{SiO}}_{2}\cdot 3H_{2}O+{\text{Ca(OH)}}_{2};$$

$$3\cdot \text{CaO}\cdot {\text{Al}}_{2}O_{3}+6H_{2}O\to 3\text{CaO}\cdot {\text{Al}}_{2}O_{3}\cdot 6H_{2}O;$$

$$x\cdot Ca{\left(OH\right)}_{2}+{\text{SiO}}_{2}+nH_{2}O\to x\text{CaO}\cdot {\text{SiO}}_{2}\cdot (n+x)H_{2}O;$$

$$y\cdot {\text{Ca(OH)}}_{2}+{\text{Al}}_{2}O_{3}+nH_{2}O\to y\text{CaO}\cdot {\text{Al}}_{2}O_{3}\cdot (n+y)H_{2}O.$$

However, Palomo *et al.* [4] proposed that boron could not replace Si within the C–S–H gel structure. Therefore, the strength is mainly caused by the hydration process of lime and cement.

## 5. Conclusions

Boron waste was reused in road base in this study. The performances of lime and lime–cement-stabilized boron waste mixtures were investigated by various experimental methods. Some conclusions can be drawn based on the above analysis. They are as follows:

1. The unconfined compressive strength (UCS) of lime-stabilized boron waste (LSB) meets the strength requirements of road base when lime content is greater than 8%. Its frost resistance is very poor. Thus, lime-stabilized boron waste can only be used in non-frozen regions.
2. A lime–cement-stabilized boron waste mixture has higher compressive and tensile strengths than those of a lime-stabilized one. Drying shrinkage coefficients of lime–cement-stabilized boron waste mixtures are all less than those of lime-stabilized boron waste. Fillers such as soil and aggregate improve drying shrinkage. It is suggested that soil and aggregate are properly added in order to reduce the drying shrinkage coefficient. Frost resistance indexes of lime–cement-stabilized boron waste mixtures are all greater than 70%. Therefore, a lime–cement-stabilized boron waste mixture is suggested for frozen regions. According to the results of this study, the proportion of LCBA-1 is the most suitable for road base.
3. The hydration process of lime and cement formed the strength of a stabilized mixture. SiO_2_ and Al_2_O_3_ in boron waste is activated by CaO (or Ca(OH)_2_). Boron was not activated by lime and cement. Therefore, boron waste can be reused as filler in road base in order to solve its effect on the environment.

In summary, boron waste can be used to construct the road base directly. Cement should be used to stabilize the mixture in order to enhance its mechanical strength and frost resistance. The use of boron waste in road base will effectively reduce its impact on the environment. In the future, a trial section of road base should be constructed to verify its serviceability for different regions.

## Figures and Tables

**Table 1:** Basic properties of boron waste.

Property	Moisture Content (%)	pH	Dry Density (g/cm^3^)	Specific Surface Area (cm^2^/g)
Value	35–40	8–10	1.2–1.5	3500–4500

**Table 2:** Parameters of particle size distribution.

Property	Diameter (μm)						*C_u_*	*C_c_*
	D10	D30	D50	D60	D90	Dav		
Value	0.67	1.35	2.42	3.11	5.88	2.92	4.60	0.90

**Table 3:** Properties of the cement.

Property	Initial Setting Time (min)	Final Setting Time (min)	Soundness	Flexural Strength (MPa)		Compressive Strength (MPa)	
				3 Days	28 Days	3 Days	28 Days
Value	145	275	Up to standard	5.6	7.7	21.3	47.6

**Table 4:** Physical properties of the soil.

Property	Maximum Dry Density (g/cm^3^)	Optimum Water Content (%)	Liquid Limit (%)	Plastic Limit (%)	Plasticity Index
Value	1.86	12.6	21.3	17.4	3.9

**Table 5:** Results of lime-stabilized boron waste (LSB) (α = 0.05).

Property			Lime Content (%)					F	*p*-Value	F-Crit	Significant
			3	5	8	10	12				
OMC (%)		AV	24.4	25.6	25.8	25.9	26	8.93	0.003	5.41	Yes
		SD	0.21	0.35	0.42	0.32	0.57				
MDD (g/cm^3^)		AV	1.62	1.61	1.59	1.59	1.58	0.26	0.894	5.41	No
		SD	0.04	0.06	0.04	0.06	0.04				
UCS (MPa)	7 days	AV	NA ^a^	0.62	0.98	1.17	1.74	95.64	1.3 × 10^−6^	6.59	Yes
		SD	NA	0.03	0.04	0.06	0.15				
	28 days	AV	NA	0.66	1.14	1.37	2.11	35.08	6.0 × 10^−5^	6.59	Yes
		SD	NA	0.04	0.11	0.08	0.32				
FRI (%)	28 days	AV	NA	NA	NA	NA	NA ^b^	-	-	-	-

Notes: ^a^ Specimens include 3% lime collapsed when they were saturated in water; ^b^ All specimens were destroyed after 4 cycles.

**Table 6:** Results of the compaction test.

No.	Lime:Cement:Boron Waste:Soil/Aggregate	OMC (%)		MDD (g/cm^3^)	
		AV	SD	AV	SD
LCBS-1	8:5:26:61	19.3	0.22	1.81	0.05
LCBS-2	8:5:43:44	18.5	0.35	1.81	0.03
LCBS-3	8:5:61:26	16.4	0.43	1.80	0.02
LCBA-1	8:5:26:61	10.2	0.45	2.11	0.04
LCBA-2	8:5:43:44	15.4	0.27	1.95	0.06
LCBA-3	8:5:61:26	17.9	0.31	1.93	0.03

**Table 7:** Mechanical properties of stabilized boron waste mixtures.

No.	Lime:Cement:Boron Waste:Soil/Aggregate	ITS (MPa)	*E* (MPa)	UTS (10^−6^)
LSB	12:0:88:0	0.35	401	873
LCBS-1	8:5:26:61	0.46	407	1130
LCBS-2	8:5:43:44	0.44	410	1073
LCBS-3	8:5:61:26	0.41	422	972
LCBA-1	8:5:26:61	0.53	565	938
LCBA-2	8:5:43:44	0.5	536	933
LCBA-3	8:5:61:26	0.46	500	920
